# Direct Write of 3D Nanoscale Mesh Objects with Platinum Precursor via Focused Helium Ion Beam Induced Deposition

**DOI:** 10.3390/mi11050527

**Published:** 2020-05-22

**Authors:** Alex Belianinov, Matthew J. Burch, Anton Ievlev, Songkil Kim, Michael G. Stanford, Kyle Mahady, Brett B. Lewis, Jason D. Fowlkes, Philip D. Rack, Olga S. Ovchinnikova

**Affiliations:** 1 Center for Nanophase Materials Science, Oak Ridge National Laboratory, Oak Ridge, TN 37831, USA; belianinova@ornl.gov (A.B.); mburch@mmm.com (M.J.B.); ievlevav@ornl.gov (A.I.); songkil.kim@pusan.ac.kr (S.K.); fowlkesjd@ornl.gov (J.D.F.); prack@utk.edu (P.D.R.); 2 School of Mechanical Engineering, Pusan National University, Busan 46241, Korea; 3 Department of Materials Science and Engineering, University of Tennessee, Knoxville, TN 37996, USA; mstanfo3@gmail.com (M.G.S.); kyle.t.mahady@gmail.com (K.M.); brettlewis@gmail.com (B.B.L.)

**Keywords:** helium ion microscopy, focused ion beam induced deposition, 3D nano-printing, direct-write nanofabrication

## Abstract

The next generation optical, electronic, biological, and sensing devices as well as platforms will inevitably extend their architecture into the 3rd dimension to enhance functionality. In focused ion beam induced deposition (FIBID), a helium gas field ion source can be used with an organometallic precursor gas to fabricate nanoscale structures in 3D with high-precision and smaller critical dimensions than focused electron beam induced deposition (FEBID), traditional liquid metal source FIBID, or other additive manufacturing technology. In this work, we report the effect of beam current, dwell time, and pixel pitch on the resultant segment and angle growth for nanoscale 3D mesh objects. We note subtle beam heating effects, which impact the segment angle and the feature size. Additionally, we investigate the competition of material deposition and sputtering during the 3D FIBID process, with helium ion microscopy experiments and Monte Carlo simulations. Our results show complex 3D mesh structures measuring ~300 nm in the largest dimension, with individual features as small as 16 nm at full width half maximum (FWHM). These assemblies can be completed in minutes, with the underlying fabrication technology compatible with existing lithographic techniques, suggesting a higher-throughput pathway to integrating FIBID with established nanofabrication techniques.

## 1. Introduction

Design, high precision placement, and high throughput of 3D conductive and insulating nanostructures is attractive for many fields. These assemblies unlock complex geometry and can be created atop other structurally complex sites, otherwise inaccessible by standard lithographic methods [1,2,3,4,5,6]. Recent advances in the bottom-up design of 3D features include two-photon lithography, focused-electron-beam-induced deposition, and gallium ion beam irradiation [7,8,9,10].

Two-photon lithography is a technique capable of producing very complex nanostructures at a spatial resolution of ~140 nm [11]. By polymerizing the precursor with a laser, virtually any structure can be printed on the micron scale. Furthermore, many precursor polymers are being developed to offer material specificity for a given application [12,13].

Similar concepts are used in additive manufacturing, where layer by layer fabrication of 3D structures is possible for a variety of materials [14,15]. Here, 40–60 μm droplets of metal ink or, for features less than 10 µm, nanoparticle inks and ion solutions may be used [16]. Recently, an additive manufacturing process featuring a lithography-based approach showcasing Ni octet-lattices with 2-μm unit cells, 300–400-nm beams, and 30-nm layers has been demonstrated [17]. Direct write via electrolysis induced by electron and ion beams from liquid precursor solutions consisting of H_2_PdCl_4_ and K_2_PtCl_6_ has also been demonstrated as an approach for creating 3D Pd and Pt structures with feature sizes ~15 nm feature size when using a He ion beam [18,19].

An alternative route towards 3D nanostructures is using a gas injection system (GIS) in tandem with a charged-particle beam—an active area of research in recent years. Significant efforts went into developing topics such as nanoscale magnetic structures, freestanding copper nanostructures, precursor development (including liquid based precursors), optimization of operating conditions for working with 2D materials, simultaneous use of oxidants for enhanced deposit purity, and theory developments [20,21,22,23,24,25,26,27,28,29,30,31,32,33,34,35,36]. Fowlkes et al. recently demonstrated a duo of predictive modeling and fabrication; showcasing complex 3D nanostructures with focused-electron-beam-induced deposition (FEBID) [37]. This method is optimized for the deposition of general mesh object models defined as interconnected networks of suspended 3D nanowires—a form of 3D nanoprinting [38,39,40,41]. Electron beam induced heating has emerged as an important physical phenomenon to consider during 3D nanoprinting [42]. Recently, 3D nanoprinting using the electron beam has been extended to include the deposition of solid nanoscale object models, moving beyond the deposition of mesh object frameworks [43]. These studies [42,43] have revealed that electron beam heating can decrease the deposition rate dynamically, effectively degrading deposit quality if the heating effect is not accounted for during design. In fact, low temperature ion-induced deposition has been demonstrated as a means to increase growth rates but should also implicitly limit the negative side-effects of beam heating [44,45]. Additionally, 3D nanoprinting with automated methods to correct for deposit distortions, regardless of the origin of the unwanted deformation [46], have been demonstrated in nanomagnetic Co/Fe nanowire framework [47].

In addition to FEBID, gallium ion beam irradiation has been used for decades to deposit metals via a process referred to as focused-ion-beam-induced deposition (FIBID) [48,49,50]. Relative to FEBID, FIBID deposits are generally larger due to the increased spot size, greater secondary electron (SE) yield, and higher ion-solid energy transfer which dissociate adsorbed precursor molecules. Complex 3D structures are also possible with the gallium beam, with relatively high throughput when compared to FEBID [48,49,51]. Recently, interest in direct-write deposition of 3D conductive structures has shifted towards achieving single-digit-nanometer-scale fabrication, largely driven by the pursuit of new approaches to interconnecting architectures [5,50,52]. Gas field ion sources have been demonstrated to manufacture smaller and cleaner 3D structures [52,53,54]. While several studies discuss and demonstrate helium ion microscopy (HIM) as a nanofabrication tool for 3D structures, few studies [55] to date systematically explore the parameter space of the HIM gas flow injection system, or the interplay of deposition and milling that occurs during the fabrication process [31,54,56,57,58].

In this work, we demonstrate complex 3D mesh geometries, with features as small as 16 nm at full width half maximum (FWHM), made in a HIM with a gas flow injection system. We rely on automated methods to control the microscope and the gas injection system settings to yield 3D nanostructures as large as 300 nm made of interconnected parts. The manufacturing process is relatively quick, with each shape completing in or under one minute. Three-dimensional structure growth on conductive as well as insulating SiO_2_ substrates is possible and is demonstrated. Furthermore, we explore beam heating effects and the interplay of deposition and milling, which occurs as matter interacts with an accelerated ion beam in FIBID of free-standing structures by mapping deposition and milling regimes as a function of beam pitch and dwell time. Understanding beam heating and the simultaneous control of both milling and deposition offers the potential for higher fidelity and higher purity structures, and the ability to repair structures in situ.

## 2. Experimental Methods

### 2.1. Growth

3D platinum structures were grown on boron doped P-type silicon substrates with a Zeiss Orion Nanofab helium ion microscope (Zeiss, Peabody, MA, USA). Substrates were plasma cleaned in nitrogen plasma for 1 minute. All deposition patterns were executed using the NanoPatterning and Visualization Engine (NPVE) pattern generator produced by Fibics Inc. (Ottawa, ON, Canada). A 25 kV He^+^ beam was used at various current to control deposit dimensions. The working distance of 8.142 mm was used throughout the experiment. The helium pressure was 2 × 10^−6^ torr with a spot number 4. The HIM was used for imaging as well as FIBID. A first generation OmniGIS Gas Injection System, (GIS) produced by Oxford Instruments (Abingdon, UK), was utilized to flow MeCpPtMe_3_ into the HIM using ultra-pure (99.9995%) nitrogen carrier gas, with a 5% duty cycle. The Pt precursor was heated to 33 °C prior to use to minimize spiking of the chamber pressure. The vertical section of the pillar was grown by parking the beam for 2 ms. The chamber pressure prior to gas injection was ~3 × 10^−7^, and ~1.0 × 10^−5^ torr during the GIS operation. The GIS was at approximately 45° with respect to the ion column. All 3D structures were generated using an in-house computer-aided design (CAD) software package (ORNL, Oak Ridge, TN, USA) [37,59]. To analyze the composition of the nanostructures, energy dispersive X-ray spectroscopy (EDS) analysis was performed on a Zeiss Merlin SEM with a Bruker EDS spectrometer (Bruker, Billerica, MA, USA).

### 2.2. Data Analysis

**Figure 1:**
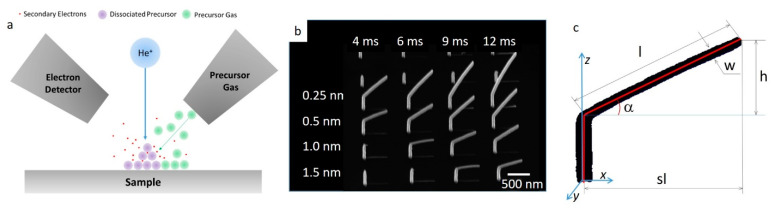
Focused-ion-beam-induced deposition (FIBID) diagram, calibration structure growth, and parametrization. (**a**) FIBID process in the helium ion microscopy (HIM) diagram, different structures are obtained by changing the beam pitch. (**b**) PtC structure array, made at 25 kV, 0.54 pA beam current with a 5 μm aperture with 8.142 mm working distance, columns are varying dwell times of 4, 6, 9, and 12 ms, and rows are varying pitch of 0.25, 0.5, 1.0 and 1.5 nm, respectively. (**c**) An example of a parametrized pillar grown for calibration of growth parameters with pillar values extracted.

Image analysis consisted of three main aspects: image pre-processing, segmentation, feature extraction, and quantification [60,61,62]. We used open-source Python 2.7 for all analysis steps. First, the image background was removed using local morphological filtering with circular filtering window of 400 pixels in diameter, to enhance the nanostructures. We then used a watershed function to automatically select the nanostructures and create a mask. After that segmentation was performed to separate all pixels corresponding to individual pillars. Finally, each pillar was fitted as by the function representing two connected linear segments. Fitting results were used to calculate length of the top segment *l*, its width *w* and angle α. An example of the single pillar analysis is shown in Figure 1c, where black dots show actual pixels of the pillar and red lines represent fitting results.

## 3. Results and Discussion

The size, deposition rate, and composition of the HIM FIBID structures with the MeCpPtMe_3_ precursor depend on many parameters. The precursor physically adsorbs onto the substrate before local dissociation by the ion beam from the generated secondary electrons (SEs) [63] as well as energy transfer via displaced deposited atoms, as illustrated in Figure 1a [64,65]. Depending on the precursor, deposition (2D and 3D), or etching are possible [66,67]. In this work, we focus on material deposition for 3D nanostructures. Firstly, we created large arrays of calibration structures at the different growth conditions shown in Figure 1b (for 0.54 pA, 25 keV), where the measured growth dimensions are illustrated in Figure 1c. The calibration structure consists of a vertical nanowire, or a pillar, which serves as the base for the subsequent deposition of a branch, or a segment, nanowire. After the calibration structures were deposited, an in-house developed analysis software was used to extract deposit dimensions and angles (see Experimental Methods) from HIM images.

**Figure 2:**
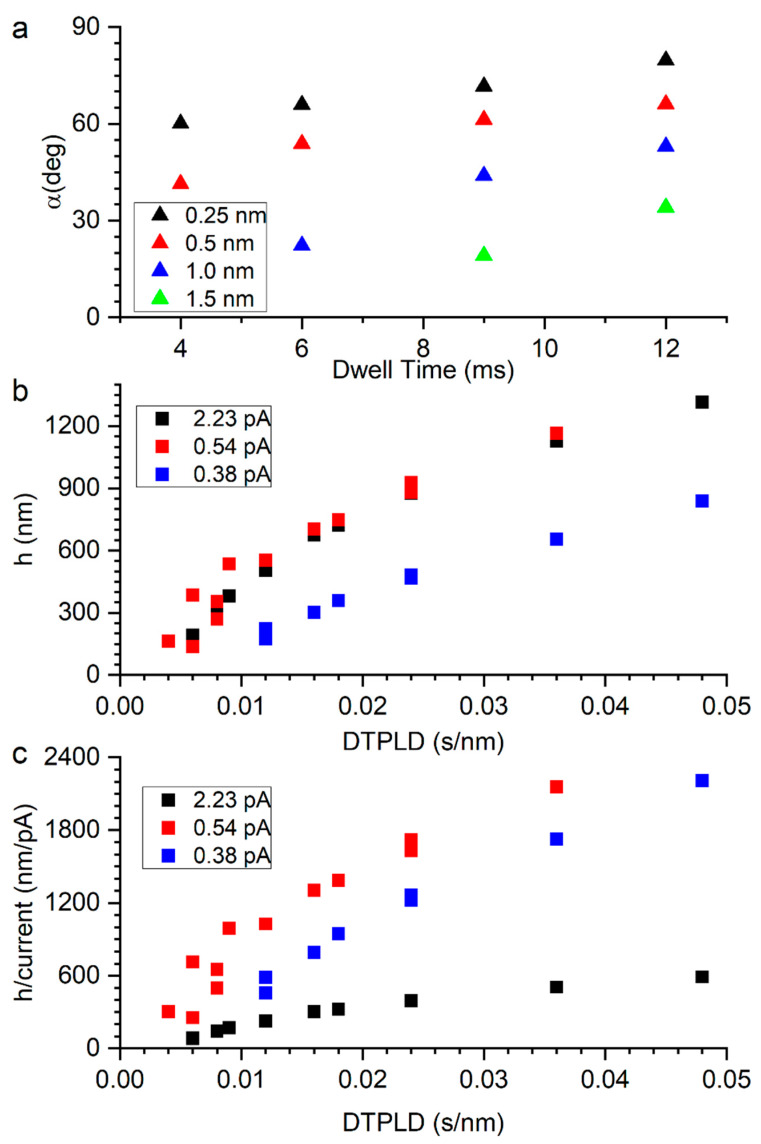
(**a**) Plot of the segment angle versus the He^+^ dwell time at different pixel point pitches for a beam energy of 25 keV, a beam current of 0.54 pA and a precursor chamber pressure of 1 × 10^−5^ Torr. (**b**) Plot of the resultant segment height (*h*) for a fixed lateral scan length (*sl*) versus dwell time per lateral displacement (DTPLD) (s nm^−1^), (**c**) plot of the vertical growth per current (nm-pA-1). Beam energy of 25 keV, beam currents of 0.38 pA, 0.54 pA and 2.3 pA, and a precursor chamber pressure of 1 × 10^−5^ torr used in (**b**,**c**).

Figure 2a is a plot of the segment angle versus the He^+^ dwell time at different pixel point pitches for a beam energy of 25 keV, a beam current of 0.54 pA, and a precursor chamber pressure of 1 × 10^−5^ torr. The plot illustrates that the segment angle increases with increasing dwell time. Furthermore, at a fixed dwell time, the segment angle also increases with decreasing pixel pitch. To illustrate the interdependence of the dwell time and pixel pitch, we combine these two terms into the dwell time per lateral displacement (DTPLD) (s nm^−1^) and plot the resultant segment height (*h* = sl*tan(α)) for a fixed lateral scan length (*sl*) versus s nm^−1^. As illustrated in Figure 2b, the data converge into a single curve. Furthermore, Figure 2b includes data for lower (0.38 pA) and higher current (2.3 pA). Note that the 0.38 pA data have a lower segment vertical growth rate (lower α), however the 2.3 pA data essentially overlay the 0.54 pA data, which show that the growth rate saturates. The data suggest that at low current the growth is limited by the beam induced dissociation (reaction rate limited), but at higher currents a transition to growth is limited by the precursor coverage (mass transport limited). Figure 2c is a plot of the vertical growth per current (nm-pA^−1^), which illustrates that the high current growth is clearly less efficient. However, it is noteworthy that the 0.54 pA efficiency is greater than the lowest current, 0.38 pA data. As illustrated below, the slightly increased current leads to an increase in the segment width (*w*) which produces a positive feedback in the vertical growth rate as more energy is deposited and secondary electrons are generated. 

Regarding the overall specific vertical growth rate (nm-s^−1^/pA^−1^), for 30 keV - 21 pA FEBID the maximum specific vertical growth efficiency maximum is ~ 5 nm-s^−1^/pA^−1^ [68]. For FIBID, because of the increased stopping power of the ion beam, the maximum deposition efficiency for the 0.38, 0.54 and 2.3 pA current 25 keV He^+^ beam is ~163 nm-s^−1^/pA^−1^, 211 nm-s^−1^/pA^−1^, and 46 nm-s^−1^/pA^−1^, respectively. 

**Figure 3:**
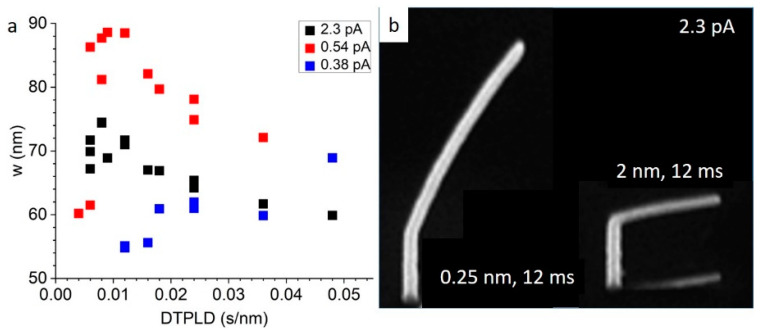
(**a**) Plot of *w* as a function of the dwell time per lateral displacement (DTPLD) (s nm^−1^), for beam energy of 25 keV, beam currents of 0.38 pA, 0.54 pA and 2.3 pA, and a precursor chamber pressure of 1 × 10^−5^ Torr. (**b**) Example of two pillars bending in segments grown at 25 keV, 2.3 pA, with 12 ms dwell time, and 0.25 nm and 2 nm pixel pitches, respectively.

Figure 3a is a plot of *w* as a function of the DTPLD for three currents. At the lowest current there is the expected increase in *w* with increasing DTPLD, which is suggestive of a reaction rate limited regime similar to a previous study [53]. At 0.54 pA, at low DTPLD, the width increases and then peaks and decreases at higher DTPLD. At high current, *w* is lower and steadily decreases with decreasing DTPLD. The decreasing *w* with increasing DTPLD and increasing current is indicative of beam heating effects and a transition from a reaction rate limited growth regime to a mass transport limited growth regime. In addition to the expected increase in heating with an increase in current, the small cross-sectional area of the resultant nanostructure also promotes a high thermal resistance, which further amplifies the heating effect. The thermal resistance increases with the increasing growth length because the substrate acts as a heat sink. A slight downward deflection in FEBID nanostructures has been identified as a signature of the onset of beam heating, which sets up a competition between higher precursor surface diffusion versus shorter precursor residence times, as the temperature progressively increases at the beam impact region [43]. Analysis of FEBID using the MeCpPtMe_3_ precursor has revealed that the residence time is the dominant parameter and thus the deposition rate decreases slightly, due to precursor depletion, and deposit bending results. Figure 3b illustrates an example of two pillars bending in segments grown at 25 keV, 2.3 pA, with 12 ms dwell time, and 0.25 nm and 2 nm pixel pitches. 

In addition to beam heating, we also postulate a secondary competitive ion beam milling effect. We investigated the sputtering process of material from grown structures using the Monte Carlo [69] simulation code EnvizION [70,71]. EnvizION simulates the ion solid interactions, similar to the SRIM [72] package, where EnvizION is coupled to a voxelized substrate, allowing cumulative sputtering and arbitrary target geometries. Each voxel represents a single atom, and the voxel size is the mean interatomic distance. The simulation allows compound target geometries [67], however, these are purely sputtering simulations and do not include the deposition process.

**Figure 4:**
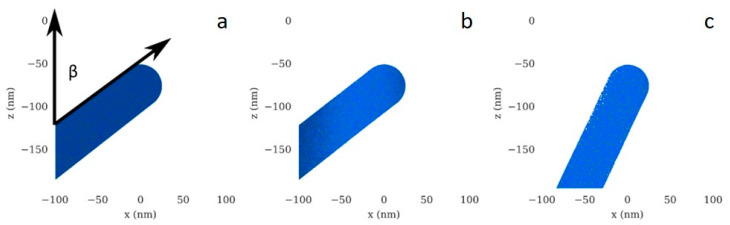
EnvizION Monte Carlo simulation results. (**a**) Schematic of the initial geometry for sputtering simulations. (**b**) Pillar corresponding to the 4 ms dwell time scan (~5.6 million ions), and (**c**) pillar corresponding to the 12 ms dwell time scan (~16.8 million ions).

The schematic for the simulated target is shown in Figure 4a, and it consists of a 25 nm radius cylindrical pillar with a cap, positioned at α degrees from the z-axis. We vary the angle α depending on the dwell time of the beam, to model the difference in pillar growth during FIBID. The experimental effect of dwell time on α is shown in Figure 2a. Initially, the simulated pillar consists of carbon and platinum with a C:Pt stoichiometry of ~8:1. A 25 kV He^+^, 50 nm FWHM Gaussian beam, with current 0.23 pA, is scanned along the pillar, from *x* = −50 nm, to *x* = 0 nm, with a pixel spacing of 0.5 nm. We simulate two dwell times: 4 ms, and 12 ms, with *β* = 52 and 25 degrees, respectively. Cross sections of the pillars after scanning are shown in Figure 4b,c. The sputter yields (corresponding to both backward and forward sputter yields) for each scan are given in Table 1. When the dwell time is 12 ms, the sputter yields are 50% higher for each specie, since the beam strikes the pillar at a glancing angle. Thus, the longer dwell time and the higher angle results in more sputtering, and when the reaction is limited by local precursor depletion the cumulative effect could contribute to narrower pillars.

**Figure 5:**
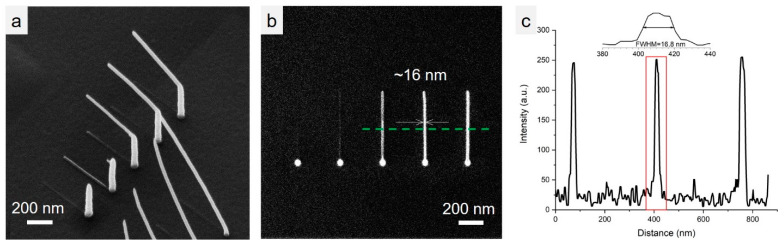
Examples of grown structures with ~16 nm features at full width half maximum (FWHM). (**a**) Side view of the structures with increasing dwell time of 4, 6, and 8 ms, respectively. (**b**) Top view of the structures in (**a**). (**c**) Line profile of the structures along the green line shown in panel (**b**) with zoom in of FWHM = 16.8 nm pillar.

**Figure 6:**
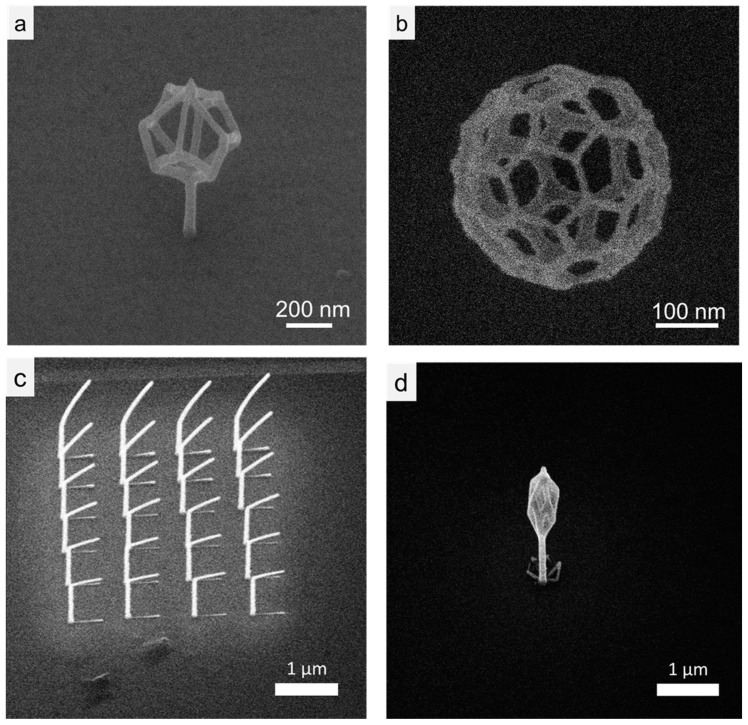
Complex 3D structures made with FIBID, (**a**) deltahedron grown on a pillar on a conductive substrate and (**b**) a truncated icosahedron a conductive substrate (**c**) pillars and (**d**) deltahedron grown on insulating SiO_2_.

We demonstrate feature sizes currently obtainable in FIBID with HIM, in Figure 5. Figure 5a is the HIM image of the branch structures, where the dwell time of the three grown posts were 4, 6 and 8 ms from left to right, with the 4 ms dwell time; producing the smallest features of ~16 nm (Figure 4c) at FWHM. These secondary processing steps with in situ feedback and control of the ion beam have the possibility to push structure fidelity down to single digit length scales and will be the focus of subsequent work. The line profile plot in Figure 5c is the overall size of the pillars, as measured across the line profile in Figure 5b. Some complex 3D geometries grown on conductive substrates with the HIM are shown in Figure 6. Figure 6a is a deltahedron grown on a post and Figure 4b is a truncated icosahedron with a total feature size of ~300 nm, which is half the total structure size to a similar icosahedron demonstrated with FEBID by Fowlkes et. al. of ~600 nm, as well as smaller than additive manufacturing approaches [18,38]. In addition to a reduction in total structure size, the nanowire thickness is nominally 20% smaller for the FIBID nanostructure in Figure 6b when compared with the complementary nanostructure deposited by FEBID [37]. However, the FIBID deposit exhibits more nanowire broadening on underlying nanowires, caused by transmitted and partially scattered primary ions emitted during the deposition of the topmost nanowire network. Further studies will be required at fixed precursor pressures to deconvolute the contributions of electron/ion scattering and total areal dose, for FEBID versus FIBID, to fully explain the origins of the broadening phenomenon.

**Figure 7:**
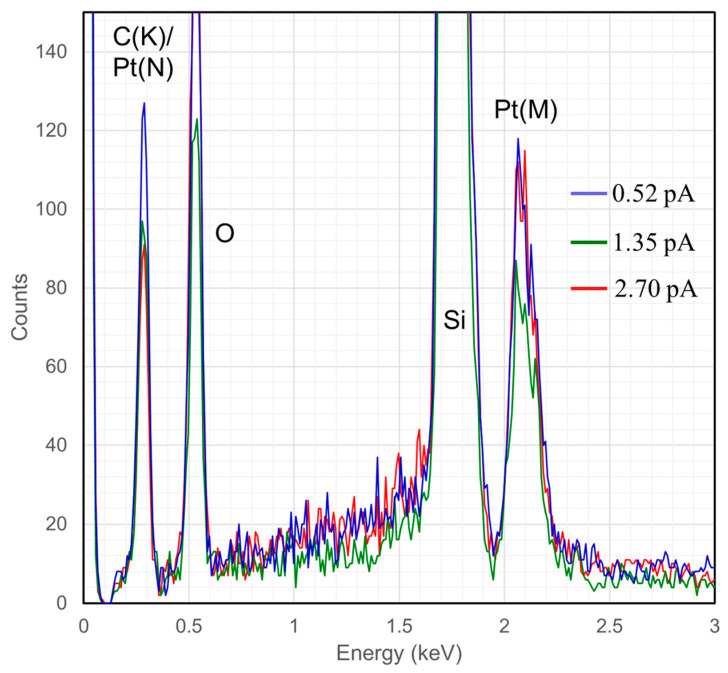
Energy dispersive X-ray spectroscopy (EDS) purity analysis of FIBID structures at three different currents. Blue = 0.52 pA, Green = 1.35 pA, Red = 2.70 pA.

Furthermore, Figure 6c,d show pillars and a deltahedron grown on SiO_2_. To estimate the composition of the as-grown FIBID 3D nanostructures, energy dispersive x-ray analysis was used to characterize a 3D “sheet”. Qualitative EDS was used to determine the uncorrected C/Pt peak ratio for a 3D deposit based on the integration range of 180–350 eV for carbon (K) and 1940–2250 eV for platinum (M), Figure 7. The C/Pt integrated EDS peak ratio of ~0.09 serves as a ‘pure Pt’ reference where small platinum (N) peak exists in the carbon peak region. The C/Pt peak ratio will vary depending on the EDS hardware but seems to vary over no more than ~0.01 [73,74,75]. This method, previously used by Mehendale [73] and Plank [74], yields an underestimate of the Pt content because the Si_L_ peak also slightly overlaps the C_K_ range. A C/Pt ratio of 0.44 ± 0.08 was calculated for our deposits. This estimate can be placed in the context of focused-electron-beam-induced deposition, or FEBID, using the same precursor MeCpPtMe_3_. The typical uncorrected C/Pt range for FEBID spans 0.7–1.3 [76], with PtC_x_ values ranging from PtC_4_ to PtC_8_. Thus, the 3D FIBID deposits appear to have PtC_x_ values where *x* < 4. For reference, 2D He-IBID deposits conducted with a beam energy of 30 kV, an exposure dose of 2 nC/µm^2^, a 6 pA beam current, and a MeCpPtMe_3_ reservoir temperature of 30 °C yielded reported deposit compositions of 15–19% Pt [76].

While not the focus of this initial study, we note in Figure 6 that collateral deposition is more pronounced on the SiO_2_ substrate versus the degenerately doped Si. Several conditions could contribute to this observation, namely, higher precursor concentration on the SiO_2_ substrate or enhanced dissociation probability on the SiO_2_. Regarding the enhanced precursor concentration, higher precursor residence time (binding energy) and/or higher surface diffusivity would lead to higher concentration in the beam interaction region. Regarding the enhanced dissociation, as was recently illustrated for ion beam induced reactive etching [77], energy transfer from incident and recoiled atoms as well as secondary electrons (SE) contribute to focused ion beam stimulated reactive processes. Naively, one would expect energy transfer in silicon would be higher as the average atomic number (14) is higher than SiO_2_ (10) and the densities comparable (Si = 2.32, SiO_2_ = 2.27 g/cm^3^). Channeling could be responsible for the decreased energy transfer in the single crystal silicon as ions scattered into open directions have much lower scattering. Regarding the secondary electron contribution, it should be noted that enhanced XeF_2_ chemical etching in Ga^+^ and Ne^+^ is dominated by the ion beam energy transfer and SE’s only contribute only a small fraction (<10%) of the chemically assisted etching. Regarding the ion induced secondary electron generation process, for conducting materials inelastic scattering of electrons is the operative mechanism [71], whereas for insulating materials, scattering from optical phonons is operative [78]. Future work will investigate the roles of these and other contributions to the direct and collateral focused ion beam induced deposition.

In conclusion, we demonstrate a novel method to grow platinum rich 3D structures atop a wide variety of substrates using a FIBID process in a helium ion microscope. Our workflow allows the users to rapidly optimize the experimental conditions for processing parameters using advanced data analytics. Three-dimensional calibration curves of the segment angle versus dwell time and pitch reveal the two variables converge to a single variable, the DTPLP. Slight deviations at DTPLP and high current suggest beam heating effects are operative in some conditions. Analysis of the segment width over a variety of patterning conditions can be understood by beam heating effects which lower the precursor coverage in the beam growth region. We illustrate this approach for fabricating complex 3D submicron architectures with minimum features measuring 16 nm, at FWHM. Future work will focus on detailed investigation of the beam heating phenomena and correlating the purity and electrical properties for different growth regimes and a wider variety of precursors.

## Figures and Tables

**Table 1:** Sputter yields of each species for the sputtering simulations of the pillars in Figure 2.

Dwell Time	C Sputter Yield (Atoms/Ion)	Pt Sputter Yield (Atoms/Ion)
4 ms	0.084	0.023
12 ms	0.15	0.035

## Notice

This manuscript has been authored by UT-Battelle, LLC, under Contract No. DE-AC0500OR22725 with the U.S. Department of Energy. The United States Government retains and the publisher, by accepting the article for publication, acknowledges that the United States Government retains a non-exclusive, paid-up, irrevocable, world-wide license to publish or reproduce the published form of this manuscript, or allow others to do so, for the United States Government purposes. The Department of Energy will provide public access to these results of federally sponsored research in accordance with the DOE Public Access Plan (http://energy.gov/downloads/doe-public-access-plan).
